# Functional Interrelationships of Microorganisms in Iron-Based Anaerobic Wastewater Treatment

**DOI:** 10.3390/microorganisms9051039

**Published:** 2021-05-12

**Authors:** Musfique Ahmed, Rifat Anwar, Dongyang Deng, Emily Garner, Lian-Shin Lin

**Affiliations:** 1 Wadsworth Department of Civil and Environmental Engineering, West Virginia University, Morgantown, WV 26506, USA; mfahmed@mix.wvu.edu (M.A.); ra0009@mix.wvu.edu (R.A.); emily.garner@mail.wvu.edu (E.G.); 2 Department of Built Environment, North Carolina A&T University, Greensboro, NC 27411, USA; ddeng@ncat.edu

**Keywords:** iron-based wastewater treatment, anaerobic treatment, functional interrelationship, Feammox, iron reducing bacteria, sulfate reducing bacteria, nitrogen fixing bacteria

## Abstract

This study explicated the functional activities of microorganisms and their interrelationships under four previously reported iron reducing conditions to identify critical factors that governed the performance of these novel iron-dosed anaerobic biological wastewater treatment processes. Various iron-reducing bacteria (FeRB) and sulfate reducing bacteria (SRB) were identified as the predominant species that concurrently facilitated organics oxidation and the main contributors to removal of organics. The high organic contents of wastewater provided sufficient electron donors for active growth of both FeRB and SRB. In addition to the organic content, Fe (III) and sulfate concentrations (expressed by Fe/S ratio) were found to play a significant role in regulating the microbial abundance and functional activities. Various fermentative bacteria contributed to this FeRB-SRB synergy by fermenting larger organic compounds to smaller compounds, which were subsequently used by FeRB and SRB. Feammox (ferric reduction coupled to ammonium oxidation) bacterium was identified in the bioreactor fed with wastewater containing ammonium. Organic substrate level was a critical factor that regulated the competitive relationship between heterotrophic FeRB and Feammox bacteria. There were evidences that suggested a synergistic relationship between FeRB and nitrogen-fixing bacteria (NFB), where ferric iron and organics concentrations both promoted microbial activities of FeRB and NFB. A concept model was developed to illustrate the identified functional interrelationships and their governing factors for further development of the iron-based wastewater treatment systems.

## 1. Introduction

Anaerobic biological treatment of wastewater has been gaining increasing attention due to its simplicity, energy efficiency, and lower sludge production, greenhouse gases emission, and capital and operational costs compared to aerobic treatment processes [1,2,3,4]. Using an anaerobic process instead of an aerobic process can reduce operating costs by approximately $160 per metric ton, and as high as $250 for some instances [5]. Carbon dioxide (CO_2_), sulfate (SO_4_^2−^), and nitrate (NO_3_^−^) are commonly used electron acceptors in anaerobic biological processes of wastewater treatment [6,7,8]. Motivated by the benefits of comanaging acid mine drainage (AMD) and municipal wastewater (MWW), cotreatment of both wastes in natural and engineering systems has previously been evaluated and showed impressive results of removing heavy metals and organic matter [9,10,11]. These studies have led to further development of innovative iron-dosed treatment processes [12,13,14]. Iron-based anaerobic treatment has multiple energy and environmental benefits including no aeration requirement, potential use of iron containing wastes, design and operation simplicity, low sludge production and CO_2_ emission, and potential resource recovery from the sludge materials [15].

As a key microbial reaction in the iron-based anaerobic biological treatment, ferric reduction is coupled to organics oxidation, in which Fe (III) is reduced to Fe (II) by receiving an electron from the organics (i.e., electron donor). As Fe(III)/Fe(II) reduction potential is comparatively higher (+0.77 V at pH 2 and +0.2 V at pH 7) than other electron acceptors (e.g., sulfate, CO_2_), iron-reducing bacteria (FeRB) can use this energy to respire a wide range of organic compounds [16]. Geobacter and Shewanella are known FeRB which were observed in most of the research on microbial iron reduction [17,18,19,20,21]. These two iron reducers have diverse ways of interacting with the ferric mineral surfaces for ferric reduction. *Geobacter* sp. is a strict anaerobe and mostly rely on pili (protein nanowires) as it does not secrete enough electron shuttling or chelating compounds [17,21]. *Shewanella* sp. has both direct and indirect electron transfer mechanisms including electron shuttles, ligands and pilin filaments. Organic composition also governs the type of FeRB present in a particular environment. For example, *Geobacter* sp. generally uses acetate and completely oxidizes it to CO_2_ while *Shewanella* sp. uses lactate as a carbon source and oxidize it to acetate [17,18].

Under substrate limiting conditions such as those found in natural environments (e.g., soil, sediments, groundwater), FeRB can outcompete sulfate reducing bacteria (SRB) for organics by diverting electron flows away from SRB [22,23,24]. In wastewater treatment applications where organic matter is abundant, both FeRB and SRB can perform carbon oxidation and concurrently contribute to the removal of organics. SRB such as *Desulfovibrio* sp. and *Desulfobulbus* sp. have been reported to facilitate incomplete oxidation of larger organic substrates (e.g., lactate) to smaller organic substrates (e.g., acetate) which could subsequently be used by FeRB [25,26]. Such symbiotic and/or competitive dynamics between FeRB and SRB are regulated by the availability of organic substrate and electron acceptors (e.g., ferric, sulfate), and associated environmental conditions such as pH and bioavailability of the electron acceptors [13].

Ferric reduction coupled to ammonium oxidation (Feammox) is another microbial metabolic function that could be used for wastewater treatment. Most Feammox studies have been conducted in natural environments such as groundwater, soils and sediments [27,28,29,30,31] and studies related to wastewater environment are extremely limited [32,33]. In strict anoxic conditions, ferric reduction has been found coupled to ammonium (NH_4_^+^) oxidation to produce either nitrogen (N_2_) (Equation (1)), nitrite (NO_2_^−^) (Equation (2)), or nitrate (NO_3_^−^) (Equation (3)) [27,29,34,35,36]. Feammox to N_2_ is energetically more favorable than Feammox to NO_2_^−^ and NO_3_^−^ under a wide range of conditions [29]. Huang and Jaffé [35,36] studied Feammox reaction in riparian wetland soils and identified *Acidimicrobiaceae bacterium A6* as the predominant bacterial species responsible for Feammox reaction.

(1) $${3\mathrm{Fe}(\mathrm{OH})}_{3}+{5H}^{+}+{\mathrm{NH}}_{4}^{+}\to 3\mathrm{Fe}\left(\mathrm{II}\right)+{9H}_{2}O+0.5\;N_{2}$$

(2) $${6\mathrm{Fe}(\mathrm{OH})}_{3}{+10H}^{+}{+\mathrm{NH}}_{4}^{+}\to 6\mathrm{Fe}\left(\mathrm{II}\right){+16H}_{2}{O+\mathrm{NO}}_{2}^{-}$$

(3) $${8\mathrm{Fe}(\mathrm{OH})}_{3}{+14H}^{+}+{\mathrm{NH}}_{4}^{+}\to 8\mathrm{Fe}\left(\mathrm{II}\right){+21H}_{2}{O+\mathrm{NO}}_{3}^{-}$$

Similar to the relationships with SRB, FeRB can potentially have symbiotic or competitive relationships with Feammox bacteria. According to redox potentials, organic carbon is a preferred electron donor compared to NH_4_^+^ and, as a result, heterotrophic FeRB can outcompete autotrophic Feammox bacteria for Fe (III) compounds. A previous study showed that only 2% of Fe(III) reduction was observed to be associated with Feammox reaction in a paddy soil when sufficient organic substrates were present [30]. Some studies reported that FeRB such as *Geobacter* can play an essential role in Feammox activities [27,37]. An indirect relationship was established between FeRB abundance and Feammox rate in these studies, as with increasing FeRB abundance Feammox reaction rate also increased. The diverse physiological characteristics of FeRB were hypothesized as the probable reason behind their contribution to Feammox activity. In treatment of organics-rich wastewater, the functional relationships between FeRB and Feammox bacteria are expected to be significantly different and the conditions in which ammonium oxidation occurs are currently not known. Moreover, in such anaerobic/anoxic environments, fermentative bacteria, nitrogen fixing bacteria (NFB), and Anammox bacteria may also be present and their functional interrelationships are largely unknown.

The objectives of this study were (i) to explicate the functional activities of various microorganisms and their interrelationships under previously reported iron dosing conditions used for wastewater treatment, (ii) to characterize the microbial diversity, abundance, and functions and to conduct comparative analyses among the different conditions, and (iii) to develop a conceptual model to illustrate the functional interrelationships of identified bacterial species and the factors that governed the microbial functions. Implications of the learned microbial diversity, abundance, metabolic functions and their interrelationships on engineering applications of the iron-dosed wastewater treatment method were discussed. 

## 2. Materials and Methods

Four bioreactors and their treatment conditions reported in previous iron-dosed wastewater treatment studies were examined. The four treatment conditions included:

1. Cotreatment of acid mine drainage and municipal wastewater (R1)
2. Fe(II)-dosed anaerobic wastewater treatment system with sludge recycling (R2)
3. Fe(III)-dosed anaerobic wastewater treatment system for organic removal (R3)
4. Fe(III)-dosed anaerobic wastewater treatment system for both organic and nutrient removal (R4)

### 2.1. Cotreatment of Acid Mine Drainage and Wastewater (R1)

Batch experiments of a two-stage process were conducted for co-treatment of field-collected AMD and municipal wastewater (MWW). In the first stage, aerobic mixing of AMD and MWW was performed to remove multivalent metals and phosphate from the AMD and MWW. In the second stage, an anaerobic attached-growth sulfidogenic bioreactor (1 L) was used to remove organics via microbial sulfate reduction. The bioreactor was operated at different COD/sulfate ratios under ambient room temperature (22 ± 1 °C), where AMD/MWW mixture pH ranging from 6.2 to 7.9, ORP values from −71 to −545 mV, and COD from 42 to 2150 mg/L. Details of this treatment design and sample analyses were presented by Deng et al. [38]. The first stage of aerobic mixing achieved significant metal removal including >97% of iron (Fe), ≈100% of aluminum (Al), and ≈75–100% manganese (Mn) removal. More than 70% chemical oxygen demand (COD) was removed in the second stage biological treatment at different COD/sulfate ratios ranging from 0.9 to 3.1. Biomass samples from the sulfidogenic bioreactor were collected for DNA extraction and other downstream analyses. The microbial DNA was amplified by polymerase chain reaction (PCR) (Eppendorf AG Mastercycler epgradient, Hamburg, Germany), and PCR amplicons of the 16S rRNA gene were cloned using TOPO TA cloning kit (Invitrogen Corporation, Carlsbad, CA, USA). The sequences were classified into taxonomic group by database project classifier, and evolutionary analyses were performed using MEGA 6 [39]. Shannon’s diversity index was calculated by using the equation H = −$\overset{s}{\underset{i=1}{\sum }}P_{i}\ln P_{i}$, where *P* is the proportion of individuals of one particular species in total number of individuals found, and *s* is the number of species.

### 2.2. Fe(II)-Dosed Anaerobic Wastewater Treatment System with Sludge Recycling (R2)

Two identical sulfidogenic attached-growth bioreactors (2.5 L each), made with acrylic cylinder and fed with continuous ferrous chloride were used to treat synthetic wastewater at different COD/sulfate and Fe/S ratios [40]. Synthetic wastewater consisted of 1.6 mM sodium acetate (C_2_H_3_O_2_Na.3H_2_O), 2.26 mM ethanol (C_2_H_6_O), 0.45 mM lactose (C_12_H_22_O_11_.H_2_O), 1.68 mM sodium bicarbonate (NaHCO_3_), and trace elements (5 mL/L influent). Ferrous iron (FeCl_2_.4H_2_O, 0.56–17.76 mM) was dosed to precipitate out sulfide to reduce the sulfide concentration in the effluent. The resultant ferrous sulfide sludge was oxidized and recycled to the bioreactors (3.5 L/d) to enhance the treatment performance. In a 510-day study period, the bioreactors were operated under room temperature (21 ± 1 °C) with incoming COD 400 mg/L, and pH ranging from 6.2 to 7.0. COD/sulfate mass ratio of 2 and Fe/S molar ratio of 1 (COD, SO_4_^2−^, and Fe (II) loading rates of 1384 mg/d, 692 mg/d and 404 mg/d) were selected for evaluating the technical feasibility of iron sulfide sludge recycling. Sludge recycling improved the COD removal to approximately 90% from the baseline performance (75%) without sludge recycling. Ferrous sulfide oxidation and recycling introduced ferric iron into the bioreactors and iron reducing condition was generated. The sludge samples and biofilms from the bioreactors and the oxidation basin were used to characterize the microbial composition. Details of the nucleic acid extraction, purification and 16S rRNA gene amplification were discussed previously [40]. Similar cloning and sequencing techniques described in the previous section for R1 were used.

### 2.3. Fe(III)-Dosed Anaerobic Wastewater Treatment System for Organic Removal (R3)

An Fe(III)-dosed anaerobic wastewater treatment system was used for COD removal from synthetic wastewater (3 mM sodium acetate anhydrous (C_2_H_3_NaO_2_), 1.54 mM ethanol (C_2_H_6_O), 0.32 mM lactose monohydrate (C_12_H_22_O_11_.H2O), 1.57 mM sodium bicarbonate (NaHCO_3_), and trace elements (4.75 mL/L influent)) with continuous ferric iron (FeCl_3_.6H_2_O, 1.32 mM, 2.50 mM, and 4.50 mM) dosing [12]. Specifically, an attached-growth bioreactor (1.4 L) made with acrylic cylinder was used to evaluate organics removal at three different Fe/S ratios (0.5, 1 and 2). The bioreactor was packed with five hundred plastic media (Evolution Aqua Ltd., UK, Kaldness K1 Biomedia, specific surface area = 500 m^2^/m^3^), resulting a working volume of 0.9 L. A consistent organic loading (COD 281 mg/d) with varied Fe(III) loadings (40, 81, and 134 mg/d), and SO_4_^2−^ loadings (197, 185, and 171 mg/d) was used to operate the bioreactor at different Fe/S molar ratios of 0.5, 1 and 2 respectively. The bioreactor was operated under ambient room temperature the pH of the bioreactor ranging from 6.5 to 7.5 and ORP from −125 mV to −250 mV. Consistent COD removal of 84–89% was observed at different Fe/S ratios. More than 90% sulfate reduction and approximately 100% iron retention were observed under all the Fe/S ratios, and both ferric and sulfate reduction played a significant role in COD oxidation. Iron retention was estimated as the total iron retained in the bioreactor and the sludge. Sludge samples from the bioreactor were collected for DNA extraction using DNeasy Powersoil DNA extraction kit (Qiagen, Germantown, MD, USA), and the 16S rRNA genes were sequenced with Illumina sequencing using bacterial/archaeal primer set 515 F/806R. Resulting reads were clustered into exact sequence variant (ESV) classifications at 100% similarity using the DADA2 platform in the QIIME2 pipeline (Qiime2-2018.4) and SILVA 16S rRNA gene database.

### 2.4. Fe(III)-Dosed Anaerobic Wastewater Treatment System for Both Organic and Nutrient Removal (R4)

Another Fe (III)-dosed attached-growth bioreactor (1.4 L) was used to evaluate the performance for concurrent organics and nutrient (N and P) removal from wastewater. The operating conditions of this bioreactor were similar to the conditions of R3 bioreactor, where the COD, N (as ammonium), P (as phosphate) and SO_4_^2−^ loading rates were 259 mg/d, 32 mg/d, 14 mg/d, and 35 mg/d respectively. Water quality analyses on the influent and effluent samples showed consistent removal of organics, ammonium, phosphate and SO_4_^2−^ from the wastewater. With a wastewater composition of COD 400 mg/L, phosphate 20 mg/L, sulfate 50 mg/L, and ammonium 50 mg/L, average removal efficiencies of COD, PO_4_^3−^-P, SO_4_^2−^ and NH_4_^+^-N was 90%, 99%, 89% and 18%, respectively. The high removal efficiency of COD was attributed to organics oxidation coupled to ferric and sulfate reduction. Biological sludge samples were collected and analyzed to investigate the presence of Feammox and other denitrifying bacteria in the bioreactor. DNA was extracted from sludge samples using the FastDNA SPIN Kit for Soil (MP Biomedicals, Solon, OH, USA). Quantitative PCR (qPCR) analyses were conducted via a QuantStudio 3 (Applied Biosystems, Thermo Fisher, Waltham, MA, USA) using previously published primers. Primer set acm342f-439r was used to target the 16S rRNA gene of Acidimicrobiaceae bacteria [36] and primer set NirS3F/NirS5R, NirK1F/NirK5R [41], and Amx368f/Amx820r [42] were used for denitrifying functional genes (*nir*S and *nir*K), and Anammox bacteria respectively. For target gene quantification, each qPCR mixture (10 µL) was composed of 5 µL of PowerUP SYBR Green Master Mix (Life Technologies, Waltham, MA, USA), 0.8 µL at 5 µM of each primer and 1 µL DNA template. Thermal cycling conditions for quantifying target genes were: 50 °C for 2 min, 95 °C for 10 min, and 45 cycles of 94 °C for 5 s, 30 s at each gene’s respective annealing temperature, and 72 °C for 30 s. The following annealing temperatures were used: 58 °C for Acidimicrobiaceae, 57 °C for *nir*S, 55 °C for *nir*K, and 56 °C for Anammox bacteria. All target genes were quantified in triplicate reactions and run on a 96-well plate with a triplicate negative control and a standard curve consisting of seven serially diluted triplicate target DNA standards, synthesized by Integrated DNA Technologies (Newark, NJ, USA). 

## 3. Results and Discussion

### 3.1. Microbial Diversity

**Figure 1:**
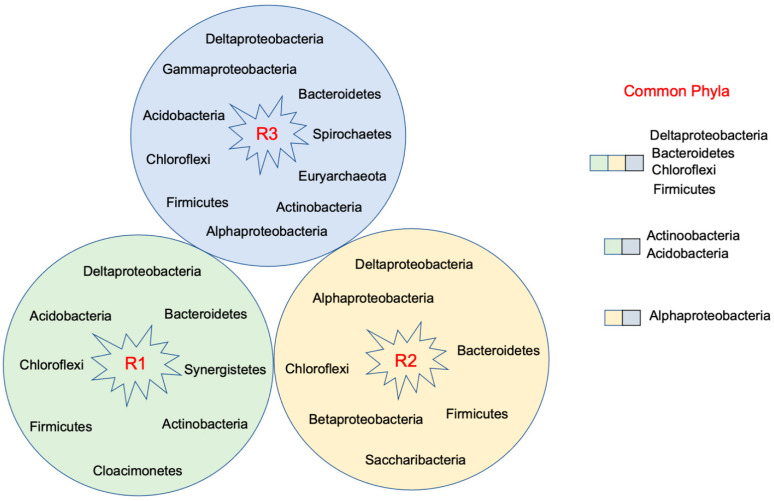
Phyla identified in R1, R2, and R3.

Various phyla were identified in bioreactors R1, R2, and R3, which depict the diverse microbial compositions in all the settings (Figure 1). Deltaproteobacteria, Alphaproteobacteria, Acidobacteria, Chloroflexi, Firmicutes, Bacteroidetes, and Actinobacteria are the common phyla that were observed in all the bioreactors. The bioreactor with Fe (III) iron dosing (R3) had higher microbial diversity (ten phyla) than R1 (eight phyla) and R2 (seven phyla). This was reflected in estimated diversity index Shannon’s H which ranged from 3.26 to 3.34 for the Fe (III)-dosed bioreactor (R3) and from 1.24 to 1.68 in the cotreatment bioreactor (R1). The higher diversity in R3 can be attributed to the high ferric dosing and prevalence of FeRB whereas R1 and R2 were mostly sulfidogenic. R1 was used to treat AMD/MWW mixtures that had high sulfate and low ferric concentrations after the first stage treatment. R2 was dosed with ferrous iron and had only limited ferric iron from the recycled oxidized sludge. We did not use microbial data of R4 reactor for diversity analysis, as this reactor was designed to investigate Feammox activities.

### 3.2. Iron-Reducing Bacteria

No FeRB were characterized for R1 as that was not the scope of that study. The only FeRB observed in the Fe (II)-dosed bioreactor (R2) was *Alkaliphilus metalliredigens.* This species is an alkaliphilic bacterium that uses lactate, acetate, and hydrogen as electron donors for Fe (III) reduction [43]. The synthetic wastewater used in the study contained primarily acetate and lactose, and had an alkalinity of 1.68 mM, which was conducive to the prevalence of this bacteria. This strictly anaerobic bacteria from the Firmicutes phylum has the capability to thrive under extreme alkaliphilic and salinity conditions [44]. 

Major putative FeRB observed in the Fe (III)-dosed bioreactor (R3) were *Geobacter* sp., *Geothrix* sp., and *Ignavibacteria* sp. Among the three FeRB, *Geobacter* sp. was predominant in abundance (83%) and others included *Geothrix* sp. (2%), and *Ignavibacteria* sp. (15%). *Geobacter* is heterotrophic, gram-negative, non-spore-forming, curved rod-shaped bacteria belonging to the Geobacteraceae family in the Deltaproteobacteria phylum [45,46,47,48,49]. This bacterium maintains an obligately anaerobic lifestyle, and typically performs complete oxidation of small organic substrates such as acetate to CO_2_ via ferric reduction. The dominance of *Geobacter* sp. in the Fe (III)-dosed bioreactor (R3) is attributed to the acetate (approximately 250 mg/L) as one of the main organic compounds of the synthetic wastewater. Acetate is one of the prime volatile fatty acids (VFAs) present in the real wastewater, which comprises approximately 49% to 71% of the total influent VFAs in full-scale wastewater treatment plants [50,51]. There are also evidences that *Geobacter* sp. can use lactate and ethanol via Fe(III) reduction [45,49].

Similar to Geobacter, Ignavibacteria has also been observed to grow well in acetate amended incubations [52]. This strictly anaerobic, moderately thermophilic, neutrophilic and obligately heterotrophic bacterium has recently been isolated from several hot springs under iron-reducing conditions [53,54]. Genome analysis of Ignavibacteria revealed it as a versatile bacterium which has the capability to live under both oxic and anoxic conditions by using a variety of electron donors and acceptors [55]. With the complex composition of real wastewater containing different types of electron donors and acceptors, presence and growth of Ignavibacteria can be anticipated. As ferric compounds are typically insoluble in the bioreactor at circumneutral pH, Geobacter and Ignavibacteria can facilitate the ferric reduction either by direct contact with outer-membrane cytochromes or via conductive pili structures [17]. For *Geobacter* sp., direct electron transfer to Fe(III) mostly occurred at the outer cell surface through c-type cytochromes [47,56,57]. Among these outer membrane (OM) cytochromes, only four of the cytochromes (OmcB, OmcS, OmcE, OmcZ) were identified to play a role in Fe (III) reduction. Another means of electron transfer for *Geobacter* sp. is to utilize Type IV pilin filaments, which are also known as ‘bacterial nanowires’ or ‘protein nanowires’ [58]. These filaments are composed of multiple copies of PilA proteins. Due to the high electrical conductivity of Geobacter pili, *Geobacter* sp. was observed to generate the highest electrical current density among exoelectrogenic bacteria [59]. This bacterial species has the potential to be used in bioelectrochemical systems for electricity generation from wastewater and/or sewage sludge to enhance energy efficiency of the iron-dosed treatment method. *Geothrix* sp. is phylogenetically different than *Geobacter* sp., but has several physiological similarities with members of the *Geobacteraceae* [48]. In addition to Fe (III), *Geothrix* sp. can utilize other electron acceptors such as Mn (IV), nitrate, fumarate, and disulfonate for redox reactions, which is also a common trait observed in the Geobacteraceae family. However, the electron transfer mechanism of *Geothrix* is different from Geobacter and Ignavibacteria. *Geothrix* sp. has the ability to facilitate iron reduction without direct contact with the insoluble Fe(III) compounds by releasing compounds that act as electron shuttles and solubilize Fe(III) from Fe(III) oxides [56].

In addition to chemical characteristics, other environmental factors may affect the growth of FeRB and SRB. Table 1 summarizes the potential growth conditions of pH and temperature for FeRB and SRB previously reported in the literature. In particular, bacteria such as *Geobacter* sp. and *Alkaliphilus metalliredigens* can grow in a broader range of temperature, making them more resilient to temperature variations than other species and adaptable for broader waste treatment applications.

### 3.3. Sulfate-Reducing Bacteria

**Figure 2:**
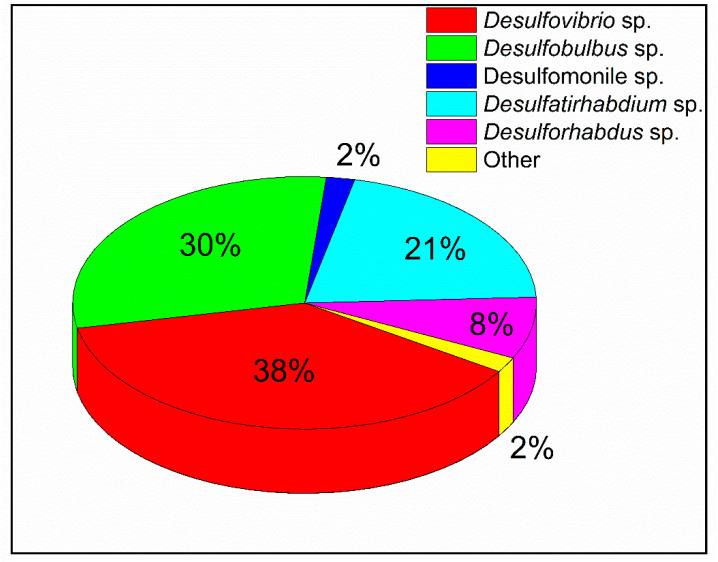
Abundance percentages (%) of different sulfate-reducing bacteria.

Putative SRB observed in the cotreatment bioreactor (R1) were *Desulfovibrio* sp., *Desulfovirga* sp., *Desulfobulbus* sp. and *Desulfatibacillum* sp.; in Fe (II)-dosed bioreactor (R2) was *Desulfomonile tiedjei*; and in Fe (III)-dosed bioreactor (R3) were *Desulfovibrio* sp., *Desulfobulbus* sp., *Desulfatirhabdium* sp., *Desulforhabdus* sp. and *Desulfomonile* sp. The microbial analysis of R3 bioreactor revealed that the major SRB was *Desulfovibrio* sp. with an abundance of 38% among the total SRB (Figure 2). Other SRB such as *Desulfobulbus* sp., *Desulfatirhabdium* sp., *Desulforhabdus* sp., *Desulfomonile* sp. were present in the bioreactor with abundances of 30%, 21%, 8% and 2% of the total SRB, respectively. 

All these SRB belong to the *Deltaproteobacteria* phylum and use sulfate as an electron acceptor for redox reactions. *Desulfovibrio* sp., *Desulfobulbus* sp., *Desulfovirga* sp., and *Desulfomonile* sp. can facilitate incomplete oxidation of large organic compounds (e.g., lactate), and *Desulfatirhabdium* sp., *Desulforhabdus* sp. can oxidize smaller organic substrate such as acetate and ethanol [25,26,60,61,62]. Co-existence of these bacteria suggests a synergistic relationship among these diverse SRB where *Desulfovibrio* sp., *Desulfobulbus* sp., and *Desulfovirga* sp. yield smaller substrates such as acetate through lactate oxidation, that can subsequently be used by other FeRB and SRB for complete oxidation of organic substrates. As wastewater is a complex mixture of various organic compounds, a diverse composition of different SRB is anticipated in iron-reducing treatment systems. 

The suitable temperature ranges for the growth of SRB (Table 1) indicate that most of the SRB can survive at the temperatures commonly found in wastewater treatment. Similarly, the pH conducive to the growth of these bacteria overlap the pH range typically observed with wastewater effluents (6.5–8.5) [63]. 

**Table 1:** Potential growth conditions pH and temperature for different FeRB and SRB observed in the bioreactors.

	Bacteria	Temperature	pH	Reference
Iron Reducing Bacteria	*Geobacter* sp.	4–37 °C	6.5–7.5	[64]
	*Ignavibacteria* sp.	30–55 °C	6.5–8.0	[53]
	*Geothrix* sp.	35–40 °C		[48]
	*Alkaliphilus metalliredigens*	4–45 °C	7.5–11.0	[65]
Sulfate Reducing Bacteria	*Desulfovibrio* sp.	15–45 °C	5.0–8.0	[66,67]
	*Desulfobulbus* sp.	10–40 °C	6.1–7.5	[68,69]
	*Desulfovirga* sp.	20–36 °C	6.6–7.4	[62]
	*Desulfatirhabdium* sp.	15–37 °C	6.5–8.0	[60]
	*Desulforhabdus* sp.	25–45 °C	6.6–8.5	[70]
	*Desulfomonile* sp.	30–38 °C	6.5–7.8	[71]
	*Desulfatibacillum* sp.	15–40 °C	6.6–7.8	[72]

### 3.4. Synergistic Relationships between FeRB and SRB

**Figure 3:**
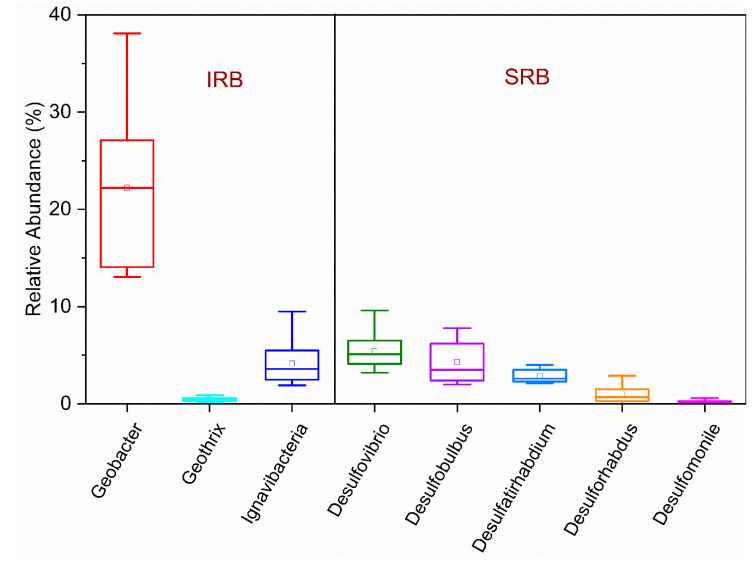
Average abundances of different FeRB and SRB in the Fe (III)-dosed bioreactor (R3).

A critical research question regarding the iron-based wastewater treatment is whether FeRB and SRB can perform synergistically to oxidize organics. Previous studies suggested that FeRB could inhibit SRB by competing for electron donors when the organic level is low [24,73,74]. In wastewater treatment applications, high organic content of wastewater can provide sufficient organic substrates and support the growth of both FeRB and SRB. The microbiological analyses of R3 showed the presence of diverse FeRB and SRB, and chemical analyses also corroborated that ferric and sulfate reduction contributed concurrently to organic oxidation [12]. Fe (III) and SO_4_^2−^ concentrations (expressed by Fe/S ratio) played a significant role in regulating the activities of FeRB and SRB in the Fe (III)-dosed treatment. The overall organic oxidation rate is dependent on the individual oxidation rates of FeRB and SRB and their populations. The average abundances of putative *Geobacter* sp. and *Ignavibacteria* sp. at different Fe/S ratios (molar ratios: 0.5, 1, and 2) were 22 ± 9%, and 4 ± 2% respectively, and those of *Desulfovibrio* sp., *Desulfobulbus* sp. and *Desulfatirhabdium* sp. were 5 ± 2%, 4 ± 2%, 3 ± 1%, respectively (Figure 3). *Desulfovibrio* sp. and *Desulfobulbus* sp. are known to facilitate incomplete oxidation of larger organic compounds to smaller compounds, which can subsequently be utilized by FeRB (*Geobacter* sp. and *Ignavibacteria* sp.) for complete oxidation, a synergy between FeRB and SRB that occurred in the Fe (III)-dosed bioreactor (R3).

**Figure 4:**
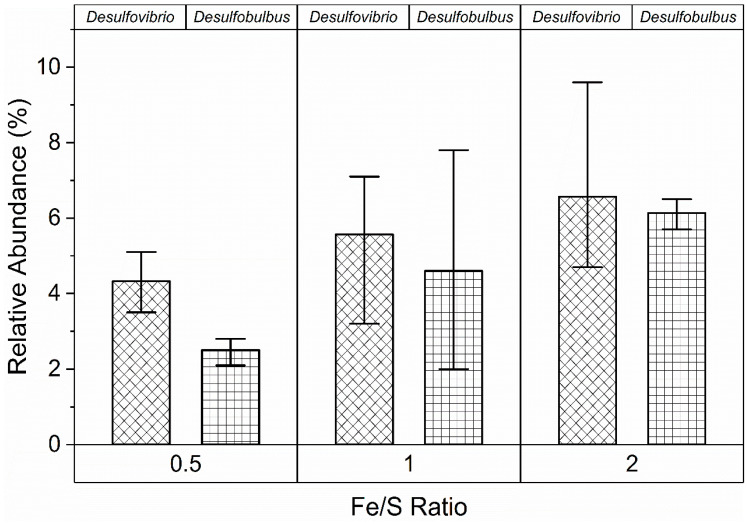
Microbial abundances of *Desulfovibrio* sp. and *Desulfobulbus* sp. at different Fe/S molar ratios in the Fe (III)-dosed bioreactor (R3).

An interesting trend observed with R3 was that the abundances of FeRB and SRB both increased with increasing ferric concentration. In this comparison, Fe/S molar ratios 0.5, 1 and 2 were used by changing Fe (III) and SO_4_^2−^ concentrations to maintain the same total equivalent of electron acceptors for all the ratios (Figure 4). While sulfate concentration decreased slightly with the increasing Fe/S ratio, the abundance of putative SRB increased from 12% to 16%. This was attributed to presence of *Desulfovibrio* sp. and *Desulfobulbus* sp. which have been reported capable of facilitating both ferric and sulfate reduction under iron reducing conditions [75,76,77]. For examples, sulfate reducers were reported to produce H_2_S via sulfate reduction, which can chemically reduce Fe (III) oxyhydroxides to form iron sulfides [78]. There is also evidence that these SRB could reduce Fe (III) directly through an enzymatic Fe (III) mechanism and produce siderite concretions [75]. These synergistic relationships between FeRB and SRB under the iron-reducing conditions in R3 can be an important microbial feature that contributes to the resilience of the iron-dosed biological treatment. 

### 3.5. Feammox and Denitrifying Bacteria

R4 was designed to investigate the presence and activities of Feammox bacteria in the bioreactor when organic substrate was not limited. The microbiological analysis showed the presence of Acidimicrobiaceae bacterium at a concentration of 1.84 × 10^6^ gene copies/mL. This *Acidimicrobiaceae* sp., (represented by band A6 by Huang and Jaffe) belongs to the *Actinobacteria* phylum, which is the only representative of Feammox bacteria [35]. This *Acidimicrobiaceae A6* is a Gram-positive, rod-shaped bacteria with an average length of 1.5–3 μm. Approximately 18% removal of NH_4_^+^-N with significant presence of *Acidimicrobiaceae* sp. in R4 showed the evidence of the Feammox activity in the Fe (III)-dosed bioreactor. The disparity in the high COD removal (90%) and the low NH_4_^+^-N removal (18%) indicated the competitive advantage of heterotrophic FeRB over the Feammox bacteria for ferric iron as the common electron acceptor. However, FeRB did not entirely suppress the Feammox activity.

Another important aspect of Feammox reaction is the production of N products including nitrite (NO_2_^−^), nitrate (NO_3_^−^) or nitrogen (N_2_) from NH_4_^+^. Our results showed the insignificant presence of NO_2_^−^ and NO_3_^−^ in the effluent of R4. The presence of denitrifying functional genes *nir*S and *nir*K with the concentrations of 1.05 × 10^10^ gene copies/mL and 6.80 × 10^7^ gene copies/mL, respectively, indicated the denitrifying activities in the bioreactor. These denitrifying activities were most likely stimulated by NO_2_^−^ generated from Feammox. Due to denitrification, NO_2_^−^ and NO_3_^−^ did not accumulate in the bioreactor. As no Anammox bacteria were observed in the samples, Anammox reaction that transforms NO_2_^−^ to N_2_ was considered an insignificant microbial pathway in this Fe (III)-dosed bioreactor.

### 3.6. Fermentative Bacteria

Diverse fermentative bacteria were observed in the biomass samples of the bioreactors (R1, R2, and R3, Table 2). All these fermentative bacteria were capable of fermenting large organic compounds to smaller compounds [79,80,81,82,83,84,85,86,87,88]. These smaller organic compounds can then be utilized by FeRB and SRB for further carbon oxidation. This suggests a synergistic relationship of fermentative bacteria with FeRB and SRB for substrate utilization. The presence of such wide range of fermentative bacteria contributed to the high microbial diversity under the iron-reducing conditions in these bioreactors.

Apart from FeRB, strains of fermentative *Clostridium* were observed that are known to perform dissimilatory iron reduction [89,90]. The presence of *Clostridium* sp. in the bioreactors suggests the possibility of their contribution to Fe (III) reduction. As fermentation takes place in the absence of exogenous electron acceptors, fermentation pathway needs to produce fermentative products that can be used as electron acceptors to dispose of the electrons produced during oxidation reactions. If additional electron acceptors such as Fe (III) are present, these excess reducing equivalents (electrons) might be delivered to them. The diversion of reducing equivalents to Fe (III) might provide an energetic advantage through utilizing the oxidation of coenzyme nicotinamide adenine dinucleotide hydrogen (NADH) coupled to Fe(III) reduction to yield ATP [89] or through change in the fermentation end products.

### 3.7. Nitrogen-Fixing Bacteria

The major NFB observed in these bioreactors were members of the Pleomorphomonas genus, which are Gram-negative, nonmotile, and pleomorphic bacteria belonging to the Alphaproteobacteria phylum [91]. They have the ability to fix atmospheric nitrogen where bioavailable N becomes limiting [92]. 

A previous study on potential synergy between FeRB and NFB in flooded paddy soils showed a positive correlation between the two types of bacteria [93]. The results showed that FeRB played an important role in the microbial nitrogen-fixing process in the presence of sufficient Fe (III). With increased iron concentrations, abundance of both NFB and FeRB increased. Similar results were observed by Ahmed et al. in their batch reactors, where with increasing Fe/S ratio, abundance of *Pleomorphomonas* sp. also increased [13]. Addition of organic carbon (e.g., glucose) also resulted in significant changes in community structures of putative FeRB and NFB [93]. The FeRB-NFB synergy promoted nitrogen fixation was attributed to two potential reaction pathways. One is that some FeRB can reduce N_2_ directly to NH_3_ [94,95], and the other is that some FeRB can indirectly promote nitrogen fixation by utilizing H_2_ as an electron donor, and preventing biological nitrogen fixation inhibition by H_2_. Fermentative bacteria may add to the complexity of the FeRB-NFB synergy. For example, *Clostridium* sp. are known to produce H_2_ by fermenting larger organic compounds and presence of hydrogen-utilizing FeRB helps prevent H_2_ inhibition of NFB. Some genera of *Clostridium* have been reported to be capable of biological nitrogen fixation [96], which could meet the N demand from microbial growth under N limiting conditions. Collectively, FeRB, NFB, and fermentative bacteria may synergistically promote nitrogen fixation in Fe (III)-reducing bioreactors. 

## 4. Functional Interrelationships among Microorganisms in Iron-Dosed Bioreactors

**Figure 5:**
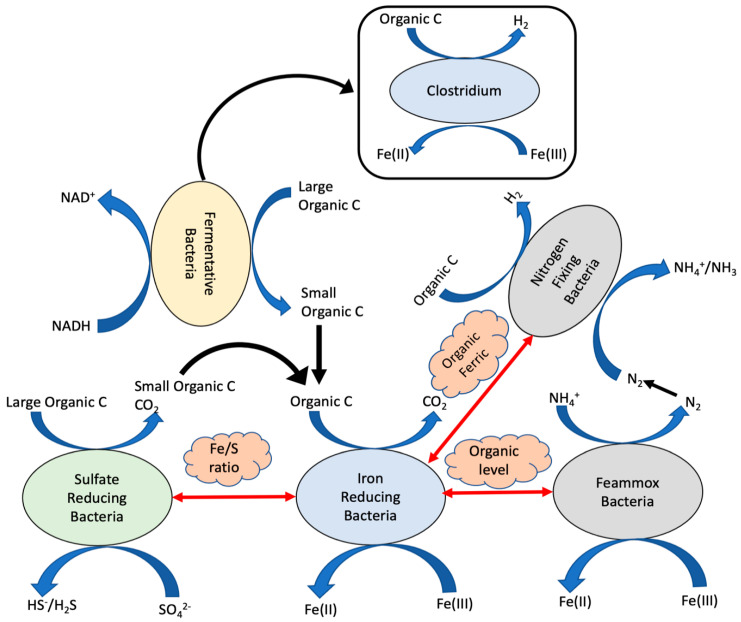
Functional interrelationships depicting microbial activities of different bacteria in iron-reducing wastewater treatment.

A conceptual model of functional interrelationships was developed based on the putative functions of the bacteria identified in the iron-dosed bioreactors (Figure 5). It illustrates the synergistic and competitive relationships among the identified bacteria and the major factors that govern the interrelationships.

Due to ubiquitous presence of sulfate in wastewater, both FeRB and SRB are the major bacterial species that contribute to organic oxidation in the iron-dosed bioreactors. Fe/S ratio (measured by Fe (IIII) and SO_4_^2−^ concentrations of the inflows) is an important operating factor that regulates the activities of FeRB and SRB. Some SRB contribute to the treatment through direct organic oxidation to CO_2_, and some SRB through partial oxidation of large organic compounds to small compounds, which are then used by FeRB. Abundances of FeRB, SRB, and other microbes are regulated by the concentrations and bioavailability of electron acceptors (e.g., Fe (III), SO_4_^2−^) and electron donors (e.g., organics, NH_4_^+^). Fermentative bacteria contribute to the treatment by facilitating conversions of larger organic compounds to smaller compounds that are subsequently used by FeRB and SRB. Fermentative bacteria such as Clostridium can also participate in direct Fe (III) reduction under iron reducing conditions. 

Our analyses suggest occurrence of the “heterotrophic vs. autotrophic” competition between FeRB and Feammox bacteria. In the presence of abundant ferric iron, organic substrate level is a key factor that regulates their activities and consequently organic and ammonium removal efficiencies. With the wastewater composition used in these studies (COD 400–420 mg/L), FeRB are the main contributor to organic oxidation and outcompete the Feammox bacteria for ferric iron, resulting in only 18% NH_4_^+^ removal. Evidences also suggest another synergistic relationship between NFB and FeRB that could occur in the iron-based treatment system. In nitrogen-limiting growth conditions (e.g., R3), nitrogen fixation by NFB is an important mechanism to meet the demand of bioavailable N of microbial growth. In the presence of ferric iron, the FeRB-NFB synergy is evidenced in the positive correlations between microbial abundance of both FeRB and NFB, and iron concentration [93,94]. Overall, this concept model provides a baseline understanding of these functional interrelationships, which is critical for further developing the iron-based treatment technologies. 

## 5. Discussion

Several microbial functional interrelationships and their governing factors in Figure 5 can potentially be used to meet various needs of waste treatment, and they are explored in this section.

The synergistic relationship observed between FeRB and SRB can be very useful for treatment of sulfate-rich wastewaters. Sulfate-rich wastewaters are generated by many industrial processes such as paper mills and the food processing industry where sulfuric acid or sulfate rich feedstocks are used [97,98]. Fe (III)-dosing to provide sufficient electron acceptor in addition to sulfate for organic removal can be an effective and energy-efficient method for managing these wastewaters. In such applications, Fe/S ratio can be used as a key operating parameter to remove organic pollutants and limit sulfide toxicity by chemical precipitation of FeS. The remaining reduced chemicals (i.e., ferrous, sulfide) in the effluent of the biological treatment can be readily oxidized in a polishing unit before environmental discharge.

Presence of Feammox bacteria in the Fe (III)-dosed bioreactor (R4) suggests the prospect of concurrent organic and ammonium removal in a single Fe (III)-dosed bioreactor. Nevertheless, as organic substrate level plays a major role in governing the competitive activities of heterotrophic iron reducers and autotrophic Feammox bacteria, design considerations need to be made for Fe (III)-dosed treatment to achieve satisfactory organic and nutrient removal. For example, Feammox activities may be intensified by adopting a two-stage treatment process, where the first stage is used to remove organic carbons, and the second stage is used for ammonium oxidation via Feammox. For nutrient-rich wastewaters containing low organic content, one-stage treatment may be sufficient to use Feammox activities for N removal. 

A thought-provoking aspect of the identified functional interrelationships is the synergy between FeRB and NFB. This synergistic relationship is augmented by both ferric iron and organic substrate, and can potentially be employed as an energy-efficient method for ammonium production. Such an engineering application would also require techniques that can recover the produced ammonium in separate process units to maintain N limiting conditions. The limiting factors of this synergism need to be identified and technoeconomic feasibility of this approach for ammonium production warrant further studies.

Microbial data of these iron-based bioreactors showed insignificant presence of methanogenic bacteria, as thermodynamically Fe(III) reduction is more favorable than the methanogenic process and generally can suppress methane production [74,99]. However, recent studies in paddy soil environments suggested syntrophic relationships between FeRB and methanogens [100,101] and postulated that bioaugmentation with iron-reducing microbial consortium can intensify methanogenic process [102]. With less bioavailable ferric, *Geobacter* species has been found to have syntrophic associations with methanogens through direct interspecies electron transfer (DIET) and thus can attribute to increased methane production [101]. This syntrophic relationship can potentially be used in anaerobic digestion processes to enhance biogas production by iron dosing. 

## 6. Conclusions

The functional interrelationships presented in this study provide insights to the various synergistic and competitive relationships in wastewater treatment under iron-reducing conditions. They are crucial for further development and design of the iron-based biological technology for optimum treatment performance. The factors governing these relationships need to be considered systematically to develop guidelines for operating the treatment process. Moreover, the interrelationships reveal that there are great opportunities to develop iron-based treatment not only for wastewater management, but also for enhanced nutrient (e.g., ammonium) and biogas production. 

## Figures and Tables

**Table 2:** Fermentative bacteria identified in bioreactors R1, R2, and R3.

Bacteria	Phyla	Functional Activities	Bioreactor
*Clostridium* sp.	Firmicutes	Ferment glucose, lactose to produce acetate and H_2_	R1, R3
*Prolixibacter* sp.	Bacteroidetes	Ferment sugar, lactose to acetate and other smaller C compounds	R1
*Marinilabilia salmonicolor*	Bacteroidetes	Ferment lactose to smaller C compounds	R1
*Leptolinea tardivitalis*	Chloroflexi	Ferment glucose, fructose, and sucrose to smaller C compounds	R1
*Ruminococcaceae bacterium*	Firmicutes	Ferment lactate to smaller C compounds	R1
*Sedimentibacter* sp.	Firmicutes	Ferment pyruvate with the presence of yeast extract to produce acetate, lactate	R1
*Candidatus Saccharimonas*	Saccharibacteria	Ferment sugars to smaller compounds	R2
*Parapedobacter* sp.	Bacteroidetes	Ferment glucose, lactose to smaller C compounds	R2
*Paludibacter* sp.	Bacteroidetes	Ferment glucose to acetate	R2, R3
*Treponema* sp.	Spirochaetes	Ferment glucose, lactose to smaller C compounds	R3
*Ruminiclostridium* sp.	Firmicutes	Ferment glucose, cellulose to acetate, ethanol, and lactate	R3
*Anaerolineae* sp.	Chloroflexi	Ferment glucose, lactose to smaller C compounds	R3

## Data Availability

DNA sequencing data used in this study are cited in the ‘Materials and Methods’ section.
